# A Phonocardiographic-Based Fiber-Optic Sensor and Adaptive Filtering System for Noninvasive Continuous Fetal Heart Rate Monitoring

**DOI:** 10.3390/s17040890

**Published:** 2017-04-18

**Authors:** Radek Martinek, Jan Nedoma, Marcel Fajkus, Radana Kahankova, Jaromir Konecny, Petr Janku, Stanislav Kepak, Petr Bilik, Homer Nazeran

**Affiliations:** 1 Department of Cybernetics and Biomedical Engineering, Faculty of Electrical Engineering and Computer Science, VSB-Technical University of Ostrava, 17 Listopadu 15, Ostrava 70833, Czech Republic; radana.kahankova@vsb.cz (R.K.); jaromir.konecny@vsb.cz (J.K.); petr.bilik@vsb.cz (P.B.); 2 Department of Telecommunications, Faculty of Electrical Engineering and Computer Science, VSB-Technical University of Ostrava, 17 Listopadu 15, Ostrava 70833, Czech Republic; jan.nedoma@vsb.cz (J.N.); marcel.fajkus@vsb.cz (M.F.); stanislav.kepak@vsb.cz (S.K.); 3 Department of Obstetrics and Gynecology, Masaryk University and University Hospital Brno, Jihlavska 20, 625 00 Brno, Czech Republic; janku.petr@fnbrno.cz; 4 Department of Electrical and Computer Engineering, University of Texas El Paso, 500 W University Ave, El Paso, TX 79968, USA; hnazeran@utep.edu

**Keywords:** interferometer, fetal heart rate (fHR), maternal heart rate (mHR), EMI-free, adaptive system, Least Mean Squares (LMS) algorithm, Normalized Least Mean Square (NLMS) algorithm, fetal phonocardiography (fPCG), maternal heart sounds (mHS), fetal heart sounds (fHS)

## Abstract

This paper focuses on the design, realization, and verification of a novel phonocardiographic- based fiber-optic sensor and adaptive signal processing system for noninvasive continuous fetal heart rate (fHR) monitoring. Our proposed system utilizes two Mach-Zehnder interferometeric sensors. Based on the analysis of real measurement data, we developed a simplified dynamic model for the generation and distribution of heart sounds throughout the human body. Building on this signal model, we then designed, implemented, and verified our adaptive signal processing system by implementing two stochastic gradient-based algorithms: the Least Mean Square Algorithm (LMS), and the Normalized Least Mean Square (NLMS) Algorithm. With this system we were able to extract the fHR information from high quality fetal phonocardiograms (fPCGs), filtered from abdominal maternal phonocardiograms (mPCGs) by performing fPCG signal peak detection. Common signal processing methods such as linear filtering, signal subtraction, and others could not be used for this purpose as fPCG and mPCG signals share overlapping frequency spectra. The performance of the adaptive system was evaluated by using both qualitative (gynecological studies) and quantitative measures such as: Signal-to-Noise Ratio—SNR, Root Mean Square Error—RMSE, Sensitivity—S+, and Positive Predictive Value—PPV.

## 1. Introduction

The monitoring of fetal heart sound by general practitioners dates back to the second half of the 17th century when these signs were considered essential to distinguish between live and dead fetuses. To listen to these heart sounds, the so-called Pinard’s stethoscope or its modern versions have been in use for several decades [1,2,3]. With general advancements in the discipline of electrical engineering and the arrival of Electronic Fetal Heart Monitoring or Electronic Fetal Monitoring (EFM), significant progress has been made in this field to date [4,5]. Currently EFM serves as a valuable diagnostic tool to detect hypoxic conditions before and during delivery [6].

Currently, the most frequently used fetal monitoring methods by clinicians are mainly centered on the ultrasound modality. For example, fetal echocardiography (fECHO) is used for diagnosis of congenital heart defects (20th–23rd week of pregnancy), and cardiotocography (CTG) simultaneously measures fetal heartbeat and maternal uterine contractions [7,8,9,10]. The current CTG technology is well advanced and it is a routine part of modern obstetrics in all developed countries. Thanks to the utilization of CTG, the mortality rate of newborn babies during delivery has decreased. However, one of the disadvantages of this method is its high sensitivity to different types of noise generated by maternal movements, which requires frequent repositioning of the ultrasound transducers. This method does not seem suitable for long-term continuous fetal monitoring due to the potential harmful effects of ultrasonic radiation on the fetus, which are not well understood at this stage. The truth is that this monitoring approach is still one of the most widely used methods in clinical practice. Some of the other main methods used for fetal heart rate monitoring are: fetal electrocardiography (fECG), fetal phonocardiography (fPCG) and fetal magnetocardiography (fMCG). Basically, fECG shows the electrical activity of the heart, fPCG describes its mechanical (acoustic) activity, and fMCG is a recording of the magnetic field of the heart [11]. A comparison of the pros and cons of individual methods is shown in Table 1.

One of the most important parameters monitored by EFM is the fetal heart rate (fHR). The fHR data can be acquired either invasively (by means of a scalp electrode inserted transvaginally secured to the fetal fontanel), or non-invasively. The invasive method produces a better quality signal (higher Signal-to-Noise Ratio—SNR), due to the fact that the heart rate is measured directly from the fetus’s head. The invasive method provides a very important tool in improving the diagnostic quality of conventional CTG as well as the validation of noninvasive ECG methods. Unfortunately, the invasive approach has a number of disadvantages. Obviously, the invasive nature of this method does not allow its utilization before delivery (before the rupture of membranes and the release of amniotic fluid). Also during delivery, it increases the possibility of infections and endangers the pregnant woman as well as her fetus. We can state with a fair degree of confidence that the current preferred trend in medicine is to use noninvasive methods [12,13,14]. Even though noninvasive monitoring is more suitable for ensuring the comfort and safety of both the fetus and the mother, fetal recordings are considerably degraded by the fetus’s surrounding influences such as technical and biological artifacts, especially by those effects generated by the mother (such as her heartbeats and uterine contractions). From a signal processing perspective, these undesirable signals must be reduced or removed. In this way, the extracted fECG signals are of diagnostic quality with minimal noise contamination so that the derived fHR information is highly accurate. The reliable separation of fECG signals from maternal recordings thus constitutes a critical signal-processing task and offers a special challenge in our research, which is highly focused on noninvasive continuous fetal heart rate monitoring by means of a PCG-based fiber optic system using two interferometric measurement sensors and an adaptive system, which are described below [15,16,17,18,19].

Conventional phonocardiography (PCG) is based on a noninvasive scanning of the acoustic signals (heart sounds generated by the opening and closure of the heart valves) by means of a microphone placed on the subject’s thorax. Therefore, the maternal PCG (mPCG) signals are acquired by placing a microphone on the maternal chest and the fetal PCGs (fPCGs) are recorded by positioning a microphone on the maternal abdomen. Here we describe, for the first time, an innovative method for extraction of fPCG signals from mPCG signals using a fiber optic sensor (based on two interferometric components) and an adaptive filtering system that implements two gradient-based algorithms (LMS and NMLS) for continuous monitoring of fHR. As such, we present a completely new and yet unused concept and technique in continuous fetal heart rate monitoring.

Some of the main advantages of such a system are due to the desirable characteristics of optical fibers and fiber-optic interferometers. Fiber-optic measurement sensors are resistant to technical artifacts such as electromagnetic interferences; therefore, they can be used in situations where it is impossible to deploy conventional EFM methods, such as during Magnetic Resonance Imaging (MRI) examination [20,21] or in wet environments. The literature shows that this sensor is demonstrably more sensitive [22,23,24,25,26] than the currently used electret microphones [27] (acoustic sensor [28,29]). These properties make fiber-optic sensors very convenient and suitable choices for the measurement of desirable (fPCG) signals. As these sensors also detect the interfering mPCG signals with very high sensitivity, it becomes essential to deploy modern digital signal processing (DSP) approaches to accurately separate fPCG from mPCG signals.

Because the conventionally used CTG Method exposes the fetus to ultrasonic radiation, it could be considered potentially harmful and invasive and therefore the safety of the fetus may become compromised in long-term monitoring [30,31]. In contrast, our noninvasive fiber-optic interferometric senor and its associated adaptive system implemented to separate fPCG from mPCG signals represent no risk to the fetus or the mother and seem very desirable for continuous fetal heart rate monitoring during different stages of pregnancy including labor and delivery. They eliminate all the potential harmful impacts of the current methods used in clinical practice.

Fiber-optic technologies, or more precisely fiber-optic sensors (FOS), are gaining increased use for biomedical purposes in a number of fast developing applications [32,33,34,35,36,37,38,39,40,41,42,43,44,45,46,47,48,49,50,51,52,53,54,55,56]. The utilization of noninvasive fiber-optic interferometric sensors for vital sign (heart rate) monitoring are described in [22,23,43,44,57,58,59,60].

The specific aim of this paper is to describe the utility of a fiber-optic interferometric sensor and an adaptive system implementing the LMS and NLMS Algorithms for the effective extraction of fHR information from maternal phonocardiographic recordings by performing fPCG signal peak detection. Adaptive systems are able to change their parameters based on actual information in the processed signal. We should also emphasis at this point that there are other powerful methods such as the Independent Component Analysis (ICA) [61,62,63], Blind Source Separation (BSS) [64,65], Principal Component Analysis (PCA) [66], Wavelet Transform Methods (WT) [67,68], and others that we could successfully apply to fHR signal extraction. These methods and their applications to fHR signal extraction will be the subjects of our future research reports. The core of an adaptive system is formed by an identification (adaptive) algorithm [69,70,71] working in real time. We chose an adaptive algorithm for the implementation of fHR signal extraction here as such algorithms have produced a successful track record in many fields of science and engineering based on their high performance and reasonable computational demands [72,73,74].

Based on the analysis of real measurement data acquired by our sensor and adaptive signal processing system, we developed a simplified dynamic signal model for the generation and distribution of heart sounds throughout the human body. In developing our signal model we were guided by the contributions made by ALMASI et al. [75,76], who have devoted great research efforts to the development of a dynamic model for generating synthetic PCG signals. Our research efforts complement theirs and enable us to generate synthetic physiological as well as pathological maternal and fetal signals [77,78,79].

Our aim here is not to fully describe the details of the models for the generation and distribution of cardiac electrical and acoustic signals in the pregnant woman’s body (such as fHR and mHR, or more precisely fPCG and mPCG signals), but to evaluate the performance of our adaptive system on synthetic data, which morphologically conform to the data acquired by means of interferometric sensors used in our research. Our dynamic signal generation model, which is based on the works reported in [80,81,82,83,84,85], allows us to continuously generate fetal and maternal electrocardiograms (fECGs and mECGs) along with phonocardiograms (fPCG and mPCG).

The role and importance of such a dynamic signal model capable of generating realistic synthetic data at this stage of our research and development cannot be taken lightly, as it is essential to carry out quantitative evaluations of the experimental results and validate the soundness of the overall system. Currently, there are no databases (Gold Standards) available for fHR signals acquired from interferometric sensors, which were used in our experiments and in testing our methods for effective fHR extraction. In addition, in the initial phases of developing any technology and its related clinical applications, performing tests on pregnant women are not considered as a suitable option. It is imperative that new technology developments and their applications to human subjects undergo comprehensive approved clinical trials and meticulous legislative processes first. Such requirements are notably strict and rightly so for pregnant women because their fetus is extremely sensitive to external energies and stimuli (such as electromagnetic radiation, mechanical pressures, change in temperature, etc.). Considering the above factors, it is evident that the utilization of synthetic data is absolutely essential in the initial phases of research and development.

## 2. Methods

### 2.1. Fetal Phonocardiography

Fetal Phonocardiography was discovered by Kergardec, Marsac and Kennedy during the 17th century [86,87]. The heart’s mechanical activity is accompanied by the generation of a variety of characteristic sounds. These sounds are associated with changes in the speed of blood flow, as well as with the opening and closing of heart valves. Phonocardiography is a diagnostic method based on evaluations of these acoustic signals (heart sounds) which are accompanied by mechanical vibrations in the heart and its vessels [88,89,90,91].

**Figure 1:**
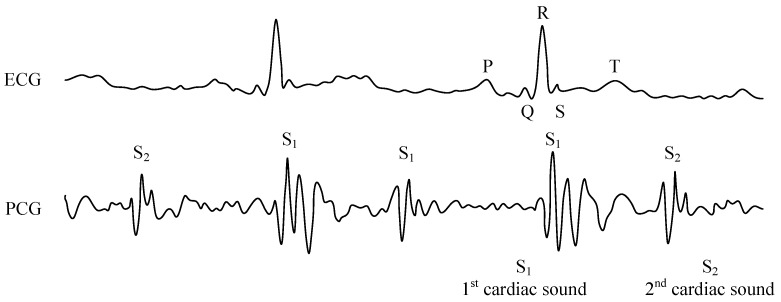
Sample recordings of electrocardiogram (ECG) and phonocardiography (PCG) signals.

Generally speaking, electrocardiograms (ECGs) and phonocardiograms (PCGs) belong to a group of basic diagnostic and monitoring measurements used to describe the cardiac electrical and mechanical activities, respectively (Figure 1). This classification is associated with the measurement technique used. ECG machines and cardiac monitors sense biopotentials through bioelectrodes placed on a test subject’s body, while PCG recordings are based on acoustic signals picked up by means of a microphone. The PCG signal is composed of two main acoustic components – the first ($S_{1}$) and the second ($S_{2}$) heart sounds. The first heart sound (systolic) is associated with the closure of the bicuspid and tricuspid valves at the beginning of the systole, and $S_{1}$ corresponds to the peak of the R wave in the ECG signal. However, the second heart sound ($S_{2}$) (diastolic) is generated by the closure of the semilunar valves, and its beginning and length are associated with the T wave on the ECG signal.

Phonocardiograms also include other heart sounds. The third heart sound ($S_{3}$) (pre-diastolic) is associated with a valve muscle quivering during fast blood flow into the valvesm while the fourth heart sound ($S_{4}$) (pre-systolic) signals a quivering valve muscle during systole in atria. $S_{3}$ and $S_{4}$ are not common for adults, and their presence is a sign of valvular insufficiency (the so-called proto-diastolic and presystolic gallop) [92,93].

In phonocardiography, different characteristics such as rate, frequency, and duration or changes in individual parts of the recorded cardiac acoustic signal are measured. Thanks to PCG and ECG signals clinicians are able to diagnose a variety of heart diseases in adults [94] and in fetuses [95] alike.

### 2.2. Non-Invasive Measurement Probe and Measurement Scheme

**Figure 2:**
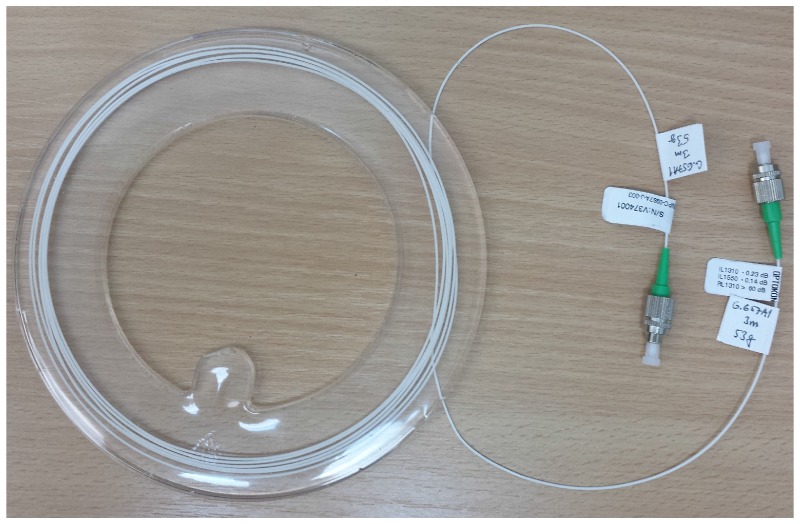
Noninvasive interferometric measurement probe.

Our noninvasive adaptive system utilizes two interferometric sensors (described in Section 2.2), which monitor the mechanical (acoustic) activity of the heart. The sensor is comprised of Mach-Zehnder fiber optic interferometers formed by 1×2 and 3×3 power couplers with an even split ratio. Interferometers belong to the highest-performance group of optical sensors as they are capable of measuring even tiny differences in the optical fiber length and the fiber core refractive index. These differences can be measured on the scale of the wavelength of the light source. The Mach-Zehnder interferometer is the most common configuration. The source is divided into two fiber arms forming the reference and the measurement paths. The measurement fiber is encapsulated into polydimethylsiloxane (PDMS) [56,96] constructing the acoustic-sensitive probe (Figure 2), while the reference fiber stays in a stable environment. The output beams then recombine at a second 3 × 3 coupler terminated in photodetectors. The output optical intensity after 3×3 coupler can be described by the following equation

(1) $$I_{n}=A_{n}+B_{n}\cos\left[\phi \left(t\right)+\phi_{drift}\left(t\right)+\frac{2\pi }{3}\left(n-1\right)\right],$$

where *n* is the coupler’s output index with a value of 1, 2 or 3, $A_{n}$ is the mean value of the optical intensity (DC component), $B_{n}$ is the optical intensity variation amplitude depending on fringe visibility, ϕ(t) is the signal of interest, and $\phi_{drift}\left(t\right)$ is the quasi-static phase shift due to coupler properties, such as dependence on hydrostatic pressure, temperature, and polarization which can be compensated during the phase demodulation step. As with any interferometric method, a demodulation algorithm is required for proper signal extraction. The use of 3 × 3 couplers enables a passive demodulation using the algorithm described in [97].

The measurements probe was encapsulated in PDMS with the designation of Sylgard 184, which is a two-component casting compound: the A component creates its own pre-polymer and the B component is a curing agent. Both components are mixed together with a weight ratio of 10:1 (A:B) according to the manufacturer’s datasheets. Bubbles and microbubbles that result from the combination of the pre-polymer and the curing agent can be removed using an ultrasonic bath. Homogeneity of the connection is achieved by using a laboratory shaker. The measurement probe contains two connectors of FC/APC type.

**Figure 3:**
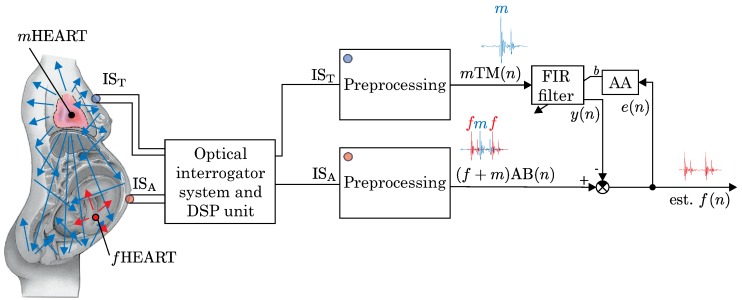
Basic schematic diagram for our noninvasive PPG-based interferometric sensors and adaptive system for fHR monitoring.

The basic scheme for the noninvasive PPG-based fiber-optic adaptive system for fHR monitoring by means of two interferometric measurement sensors placed on the chest and abdomen is shown in Figure 3. Sensors are placed on the maternal body by self-adhesive straps of 10 × 10 mm. The measurement sensor weighs 150 g and has a circular shape with a 100 mm diameter.

**Figure 4:**
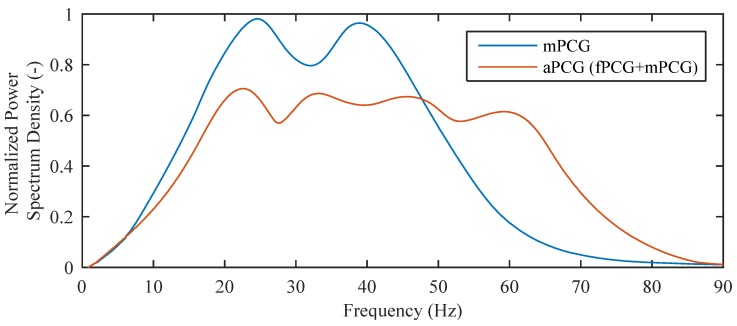
Normalized Power Spectrum Density of data acquired from the test subject.

Based on Figure 3, we can contemplate 3 approaches on how to eliminate the undesirable mPCG signal, which contaminates the abdominally recorded fPCG signal:

- **Direct Signal Subtraction**: The signal detected on the abdomen called as aPCG signal is the sum of fPCG and mPCG signals. The easiest method for removing the undesirable mPCG from the aPCG signal is a direct subtraction of the values mTM(n) from (f+m)AB(n). This approach cannot be used in practice as mPCGT measured by the thoracic fiber-optic sensor ($IS_{T}$) is not identical to the mPCGA measured by the abdominal fiber-optic sensor ($IS_{A}$). When the signal spreads from the maternal heart to the abdomen, it is influenced by different factors in the unknown body environment such as distortions due to interferences and delay caused by the signal distribution in the human body. This fact is supported by real measurements (as explained in Section 3).
- **Linear Filtering**: The next method is to use linear filtering, i.e., frequency selective filtering. However, like direct signal subtraction, linear filtering cannot effectively eliminate the undesirable mPCG signal as the desired signal (fPCG) and the unwanted signal (mPCG) share overlapping spectra (Figure 4).
- **Adaptive Filtering**: If we accept the unknown environment confined between the thoracic and abdominal fiber-optic sensors as linear, we can successfully use an adaptive filter to eliminate the fPCG signal from the aPCG signal. As an adaptive filter, the well-known FIR filter [98] whose coefficients are continuously updated by an adaptive algorithm (such as LMS or NLMS) could be used. This algorithm monitors the input and output signals from the filter, and from the error signal e(n), it tries to set filter coefficients most optimally in order to minimize the difference between the output and the required (ideal) signal. The aim of the filter is to reach a state in which the filtered thoracic mPCGT signal is the most similar to the abdominal mPCGA signal, which contaminates the fPCG signal reaching the abdominal part and whose value could be subsequently subtracted (eliminated).

Figure 3 shows the maternal and fetal hearts as two complex biosignal generators, which are mutually and acoustically separated from each other and work autonomously. From a signal processing point of view the mHEART serves as the biosource of the unwanted signal, whereas the fHEART is the biosource for the desired signal.

To further process, record, and transfer the above mentioned signals it is necessary to perform a number of operations on them, which are achieved by the Optical Interrogator System and DSP Unit. These processes (amplification, digitization, demodulation, filtering) are realized in the so-called pre-processing block. Pre-processing is realized by means of the well-known techniques, thoroughly described in technical literature, such as [22,99,100].

The amplification and digitization of the maternal and fetal signals are realized by means of the NI-USB 6210 card, which has a 16-bit Analog-to-Digital Convertor (ADC) with a built-in amplifier enabling the choice of amplification gains in steps of 1, 5, 10 and 50. The associated software support development environment of this card, LabVIEW, enables further digital processing of the data. In this application, the scanning and symmetric demodulation of data is implemented in the form of immediate variance of phase information and digital filtering.

It is assumed that the mTM(n) signal processing block produces nearly ideal (noise-free) maternal PCG signals. These are obtained by applying conventional pre-processing techniques to the $IS_{T}$ input signal. To compensate for the drift (due to fluctuations of zero isoline and breathing), we selected a lower corner frequency of 0.5 Hz. An upper corner frequency is defined especially by the phonocardiography frequency of the heart, which typically comprises frequencies ranging from 10 to 400 Hz, thus the upper marginal frequency must be set to 400 Hz [99,100,101].

In our experiments, two inputs signals were considered (since we used two interferometric sensors). The first input (reference) signal that was fed into a pre-processing block is the maternal thoracic PCG signal represented by $IS_{T}$ in the block diagram. The maternal PCG signal reflects the activity of the maternal heart (mHR). From the signal processing point of view, the mPCG signal is an unwanted signal (noise) which contaminates the desired fPCG signal. The mPCG signal is digitized by using the ADC mentioned above. In all of our experiments, we selected a sampling frequency $f_{sr}=$ 1 kHz.

The second (primary) input to the experimental system shown in Figure 3 was a signal measured in the maternal abdomen represented as $IS_{A}$, which mainly reflects the activity of the fetal heart (fHR). Here, we also used conventional pre-processing techniques to eliminate basic unwanted elements, which contaminate the (f+m)AB(n) outputs.

By using linear filtering we were able to eliminate only those signal components whose spectra did not overlap with the useful fPCG signal (provided that a predictive value was maintained).

### 2.3. Stochastic Gradient Based Adaptation

To carry out our experiments, we used an adaptive system based on the root mean square error (RMSE) criterion. Stochastic approaches require a large number of measurements to enable us to perform adequate statistics. This approach leads to the Least Mean Square Algorithm or its normalized version (LMS, NLMS), which are the basic representatives of stochastic gradient-based adaptation methods [102,103,104].

#### 2.3.1. Implementation of Adaptive LMS Algorithm

**Figure 5:**
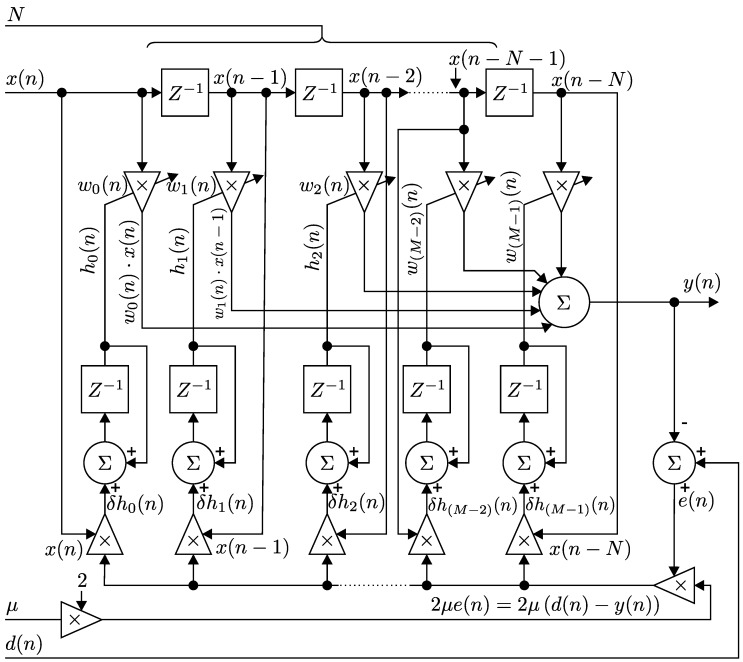
Basic scheme of an adaptive *N*-th order FIR filter with transversal structure and the LMS Algorithm.

Figure 5 shows the basic scheme for an adaptive *N*-th order FIR filter with transversal structure and implemented by using the LMS Algorithm. Each iteration of the LMS Algorithm requires the implementation of 3 different steps, respectively. First of all, a value of the output of the FIR filter y(n) is calculated according to Equation (2), valid in R:

(2) $$y\left(n\right)={\vec{w}}^{T}\left(n\right)\vec{x}\left(n\right)=\sum_{i=0}^{N}w_{i}\left(n\right)x\left(n-i\right).$$

Subsequently, a value of the estimated error signal e(n) is calculated according to Equation (3) in R, thus:

(3) e(n)=d(n)−y(n).

And finally, the values of vector scales $\vec{w}\left(n\right)$ of a relevant FIR filter are updated for the subsequent iteration according to Equation (4), valid in R.

(4) $$\begin{array}{cc} \vec{w}\left(n+1\right) & =\vec{w}\left(n\right)+2\mu e\left(n\right)\vec{x}\left(n\right),\\ \vec{w}\left(n+1\right) & =\vec{w}\left(n\right)+k_{\mu }e\left(n\right)\vec{x}\left(n\right),\\ \vec{w}\left(n+1\right)-\vec{w}\left(n\right) & =2\mu \left[d\left(n\right)-y\left(n\right)\right]\vec{x}\left(n\right),\\ & =\delta \vec{h}\left(n\right)\;\;\forall n\in Z^{+},\\ \vec{h}\left(n+1\right) & =\vec{h}\left(n\right)+\delta \vec{h}\left(n\right)\;\;\forall n\in Z^{+}. \end{array}$$

The implementation of the LMS Algorithm in R can be summarized as follows:

(5) $$\begin{array}{cc} \mathrm{BEGIN}\;\; & \vec{w}\left(n=0\right)=\vec{0}\\ \mathrm{FOR}\;\; & (n=1,2,...,N):\\ & y\left(n\right)={\vec{w}}^{T}\left(n\right)\vec{x}\left(n\right)\\ & e(n)=d(n)-y(n)\\ & \vec{w}\left(n+1\right)=\vec{w}\left(n\right)+k_{\mu }e\left(n\right)\vec{x}\left(n\right) \end{array}$$

#### 2.3.2. The Normalized Least Mean Square (NLMS) Algorithm

The Normalized LMS Algorithm (NLMS) is a variant of the LMS Algorithm with the added advantage of an accelerated convergence speed and a reasonable computational cost [105]. In this algorithm a normalized step-size $\mu_{n}$ is selected, which results in a stable and fast converging adaptation process [106].

If we consider the step size in Equation (5) to be a variable and not a constant, i.e., the equation is modified as follows:

(6) $$\vec{w}\left(n+1\right)=\vec{w}\left(n\right)+\mu \left(n\right)e\left(n\right)\vec{x}\left(n\right),$$

where the step-size can be described as:

(7) $$\mu \left(n\right)=\frac{\mu }{\delta +{\vec{x}}^{T}\left(n\right)\vec{x}\left(n\right)},$$

which means that this parameter is proportional to the inverse of the average of the total energy at the filter tap inputs. That property compensates the main drawback of the standard LMS algorithm, which is sensitive to the scaling of its input x(n):

(8) $$\vec{w}\left(n\right)=\vec{w}\left(n-1\right)+\mu \frac{e\left(n\right)\vec{x}\left(n\right)}{\delta +{\vec{x}}^{T}\left(n\right)\vec{x}\left(n\right)},$$

where μ∈(0,2] δ>0. Note that δ is the regularization parameter, which also ensures the computability of Equation (8) (prevents its denominator from becoming zero) in the case of a zero input [107].

## 3. Results

**Figure 6:**
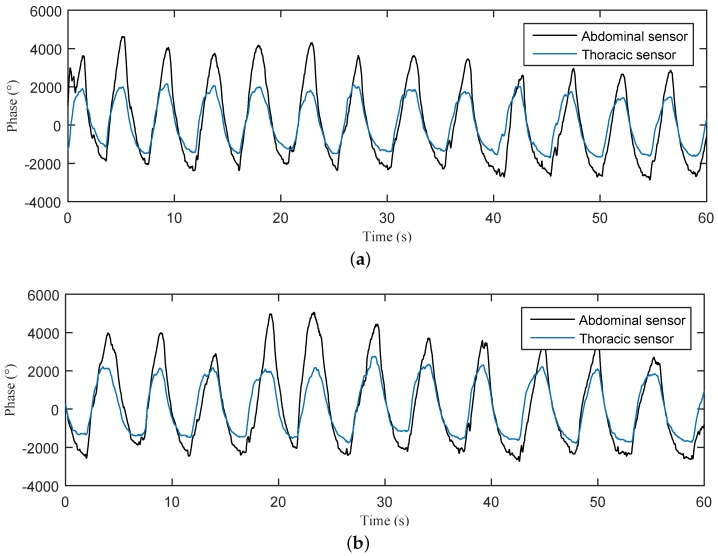
Sample plots of real raw data acquired from the thoracic and abdominal sensors of two different test subjects. (**a**) volunteer No. 1; (**b**) volunteer No. 2.

The first step in our work reported here was to model the synthetic signals ($IS_{T}$ and $IS_{A}$) based upon real measurements made from the interferometric sensors placed on the test subject’s thorax and abdomen (Figure 6). Real raw data were acquired from a group of 10 volunteer pregnant women (GA = 35–42 weeks, in a suitable research laboratory environment after obtaining their written informed consent to participate in this study. The test subjects were between the ages of 19 and 33 years, their weights were between 49 and 98 kg and their heights were between 152 and 198 cm. No significant differences were found in the quality of recorded signals based on a subject’s age, weight, and height. We captured a relevant period of signals from our designed and patented interferometric sensors. The acquired data were used as a basis for mPCG and fPCG signal model development and validation. The main objective in signal modelling was to obtain the primary signals mTM(n) from the thorax and mAM(n) from the abdomen. We dedicated substantial efforts to ensure that these synthetic signals resembled, as closely as possible, those data obtained from real measurements sensed by the abdominal and thoracic interferometric sensors. Real measurements were also necessary because there are currently no databases of mPCG and fPCG signals recorded from fiber-optic sensors, or more precisely from fiber-optic interferometers encapsulated in PDMS enclosures, which were used to satisfy the design and verification requirements of our adaptive signal processing algorithms. Based upon experimental results in our research, we have been able to create a publicly available database of maternal and fetal PCG signals, which could be used by researchers in this field to test their signal processing algorithms and validate their methods.

The novel sensor designed by our research team has yet to be approved by the relevant medical device regulatory authorities so that we would be able to carry out clinical trials on pregnant women. These regulations are extraordinarily strict for pregnant women as an unborn fetus is extremely sensitive to external factors (such as electromagnetic radiation, mechanical pressure, change in temperature, and others). Considering these practical facts, the necessity to use synthetic data in the early stages of our developmental work becomes indispensable.

The implemented fPCG and mPCG signal models used in our research are based on signals that were produced by means of our ECG signal generator and modelling of sound distribution in the human body. The models for fPCG and mPCG signals are inspired by real data acquired from interferometric sensors. The models predict that heartbeat activity causes (triggers) subsequent mechanical effects, which are distributed throughout the human body, and then reflected and attenuated after a certain period of time [108,109,110]. Our results confirm those obtained by Nagel et al. [111], who modeled maternal heart sounds (mHS) as periodic signals known as maternal pulses that originate due to blood flow sound within maternal arteries with higher amplitudes relative to fetal heart sounds (fHS).

The advantage of this conceptualization of modelling the fetal heartbeats is that it allows us to superimpose the fPCG signal on an abdominal signal, i.e., (m+f)AB(n), thus producing a primary signal suitable for experimentation with adaptive systems.

**Figure 7:**
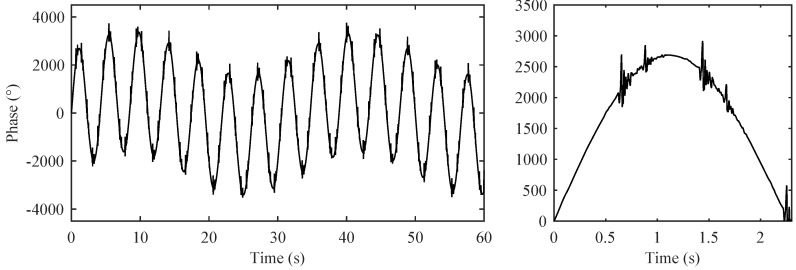
Modelled raw signal measured by $IS_{T}$ in the abdominal region (mRR ∈ <12–16> rpm; mHR ∈ <65–85> bpm). (**Left**) 60 s; (**Right**) detail in the form of 2.3 s.

Figure 7 shows the modelled raw signal ($IS_{T}$) measured on the thoracic region. Here, we can observe the breathing activity of a pregnant woman (mRR) expressed as respiration per minute (rpm). In detailed view, we can see the mPCG signal which is superimposed on the breathing activity expressed as beats per minute (bpm).

The implemented model capturing the maternal and fetal heartbeat activities shows that the fetal heart activity is decreased while dispersing through the body and is not seen in the thoracic part. This is a necessary condition for being able to use this signal for adaptive filtering. Our signal generator enables us to model the variability of respiration and heart rate activity as well [112,113,114,115]. In our experiments, we used 20-min long records of which 10 min were physiological while the remaining 10 min were pathological. Our signal generator can also model fetal gestation age from the 20th to 42nd week of pregnancy. For our experiments, we created data with a fetal gestation age (GA) of 35 weeks. The GA has a primary influence on the value of the SNR. Physiological and pathological data were modelled following the IFGO (International Federation of Gynaecology and Obstetrics) Recommendations. As we mentioned above, our sensor is more sensitive to sensing the fPCG than the mPCG signal. This property, as well as the difference between the data measured by the interferometers and conventional PCG curves, was considered when we modelled the fECG and mPCG signals for our experiments.

**Figure 8:**
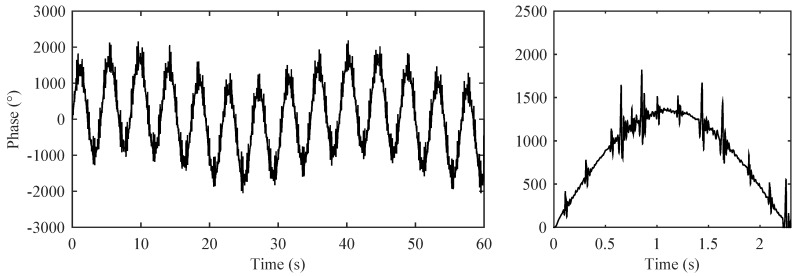
Modelled raw signal represented by $IS_{A}$ in the abdominal region (mRR ∈ <12–16> rpm; mHR ∈ <65–85> bpm, fHR ∈ <80–155> bpm, GA = 35 weeks, orientation of fetus: Right Occiput Posterior (ROP)). (**Left**) 60 s; (**Right**) detail in the form of 2.3 ss.

Figure 8 shows the modelled raw $IS_{A}$ signal in the abdominal region. Here, we can see the dominating breathing activity of a pregnant woman (mRR). In the detailed view, we can see the mPCG and fPCG signals which are superimposed on the breathing activity. The measurement clearly shows that the determination of fHR is not linear because there is a dominant maternal part mPCG in the signal. It is evident that the fPCG signal has to be obtained by means of advanced signal processing methods.

**Figure 9:**
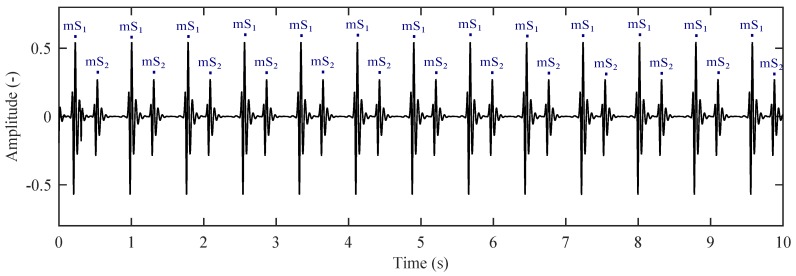
Recording of a reference signal for the adaptive system represented by $IS_{T}$ in the thoracic region.

Figure 9 shows an ideal mPCG signal after the removal of the maternal breathing artifacts (We used a second order Butterworth band-pass filter with corner frequencies $f_{L}$ = 0.75 Hz, $f_{H}$ = 400 Hz, respectively). This signal serves as a reference input signal for the adaptive system. On the basis of the obtained results we can determine mHR.

**Figure 10:**
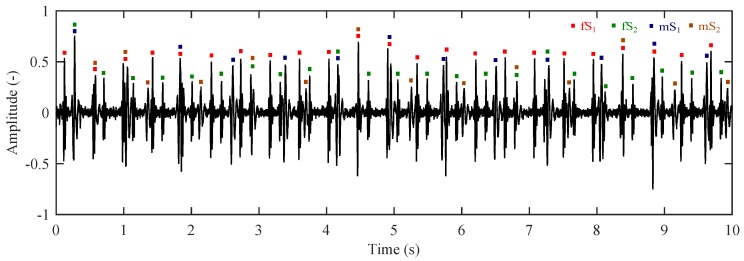
Input signal of the adaptive system formed by a mixture of maternal heart rate (mHR) and fetal heart rate (fHR).

Figure 10 shows a modelled signal measured by the abdominal sensor after the removal of maternal breathing artifacts. This signal is formed by a mixture of fPCG and mPCG signals. It is evident that the level of mPCG signal is higher than that of the fPCG signal. This measured signal does not allow us to determine the fHR without any further processing. This signal is a primary input of our implemented adaptive system.

**Figure 11:**
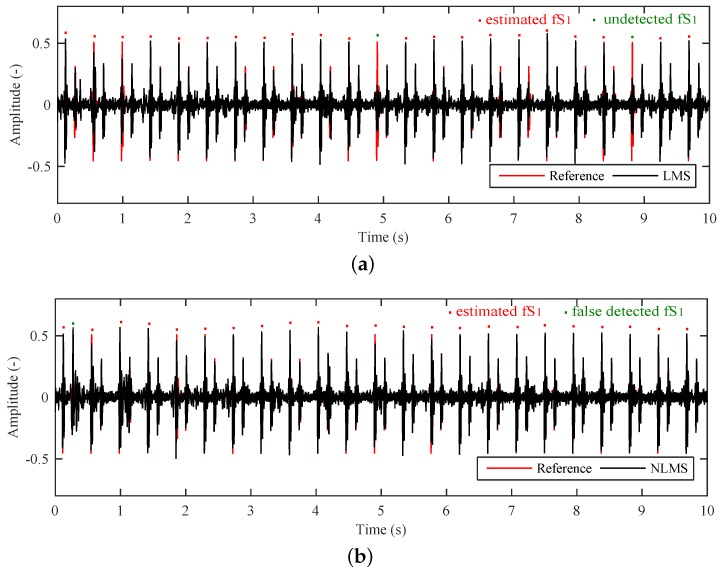
Output of the adaptive system when using (**a**) the Least Mean Square Algorithm (LMS) and (**b**) the Normalized Least Mean Square (NLMS) Algorithms.

Figure 11a shows the adaptive system output when using the LMS Algorithm and Figure 11b shows the output when using the NLMS Algorithm. The obtained results illustrate that the mPCG signal has been significantly suppressed. We observe that the elimination of the maternal component is not ideal and it is still present in the estimated signal. Nevertheless, it is attenuated well below the fPCG signal level. After this processing step, we can determine the fHR from the estimated fPCG signal by means of conventional signal processing techniques [90,116,117].

The parameters of both adaptive algorithms were set empirically based on experiments. As for the LMS Algorithm, we set the filter order *N* = 38 and the convergence constant μ = 0.1598. As for the NLMS Algorithm, an *N* = 61 with an initial μ = 0.1 were used. The primary and reference inputs of the adaptive systems were identical for both algorithms due to a corrective verification of the results.

Note the peak at the beginning of the estimated signal in Figure 11. It is caused by the fact that the algorithm is not able to converge to the optimal value (does not set the optimal step size) fast enough. The worse results of the LMS Algorithm might be caused by a poor choice of the convergence constant. In the case of the NLMS Algorithm, the value of the convergence constant varies and consequently this limitation is removed.

**Figure 12:**
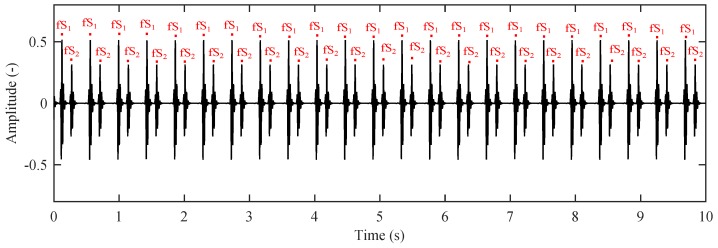
Modelled reference (ideal) signal represented by $IS_{A}$ in the abdominal region without a maternal component (fHR ∈ <80–155> bpm, GA = 35 weeks, fetus position: Right Occiput Posterior (ROP)).

Objective methods for the evaluation of filtering quality require a reference fPCG signal from which we can determine the fHR. Currently, there are no references available to represent the outputs of interferometric sensors, and thus this seems to be quite a suitable way to objectively verify the utilization of the proposed model. Figure 12 shows the reference source of the fPCG signal that enables the determination of the fHR.

**Figure 13:**
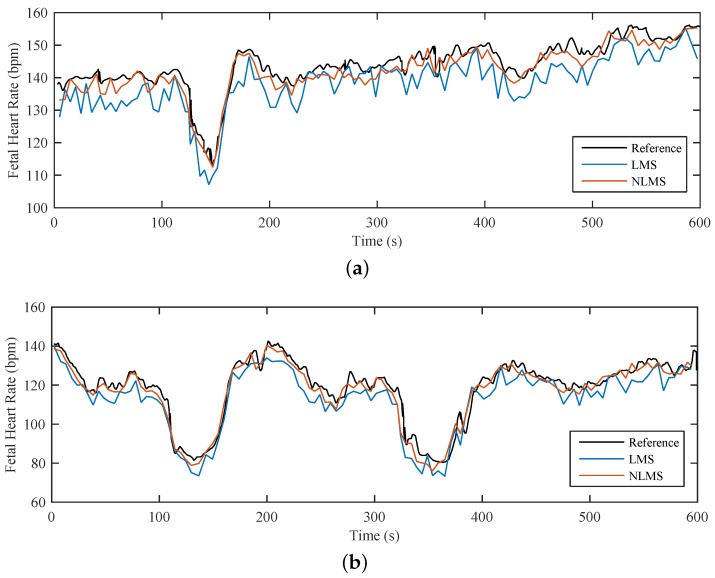
Comparison of reference and predicted time course of fHR (**a**) physiological case; (**b**) pathological case.

Figure 13a shows the reference physiological fHR time course together with its predicted tracings determined from the fiber-optic sensor and adaptive systems using the LMS and NLMS Algorithms. Figure 13b shows these time courses for a pathological fHR. Our experiments confirmed the hypothesis that filtering efficacy is influenced by neither the physiological nor the pathological states of the fHR tracings.

Figure 13 confirms that the LMS Algorithm causes a decrease in the estimated fHR values since it eliminates some of the desired fS1 sounds in addition to the undesired maternal sounds. In contrast, the fHR waveform estimated by the NLMS Algorithm does not significantly differ from the fHR reference time course and therefore it is more suitable for fHR detection.

In order to compare the differences between the reference and predicted fHRs, we made use of the Bland-Altman plots [118]. The differences between the reference and the predicted traces, ref−predic, are plotted against the average, (ref+predic)/2. The reproducibility is considered to be good if 95 % of the results lie within a ±1.96 SD (Standard Deviation) range.

**Figure 14:**
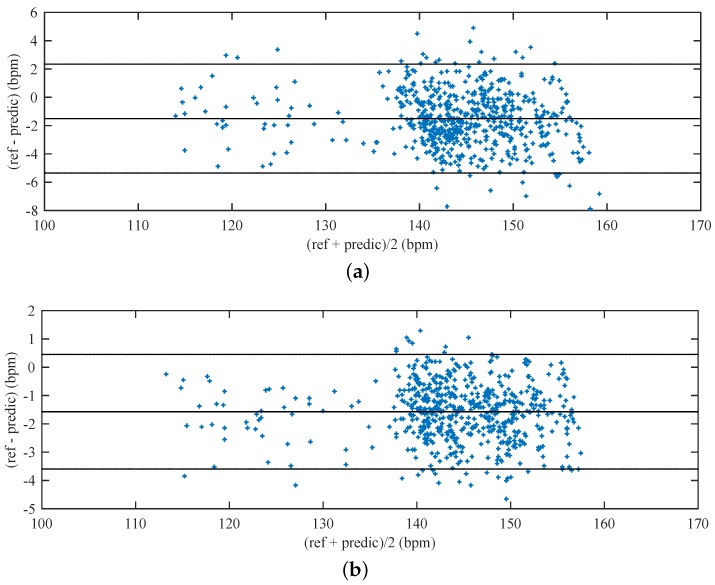
Bland-Altman statistics for reference and predicted values of fHR for (**a**) the LMS Algorithm; (**b**) The NLMS Algorithm.

Figure 14 shows the Bland-Altman statistics for reference and predicted values of fHR when using (a) the LMS; and (b) the NLMS Algorithms. The fetal heart rate is expressed in beats per minute (bpm). For the entire data set using the LMS Algorithm (608 samples), 94.74 % of the values lie within the ±1.96 SD range for the fetal heart rate determination. For the entire data set using the NLMS Algorithm (611 samples), 95.07 % of the values lie within the ±1.96 SD range for the fetal heart rate determination.

Furthermore, the Bland-Altman statistics show that the error between the reference and the predicted fetal heart rate is twice as high when using the LMS Algorithm. Possible explanations for this are described in the text above.

**Figure 15:**
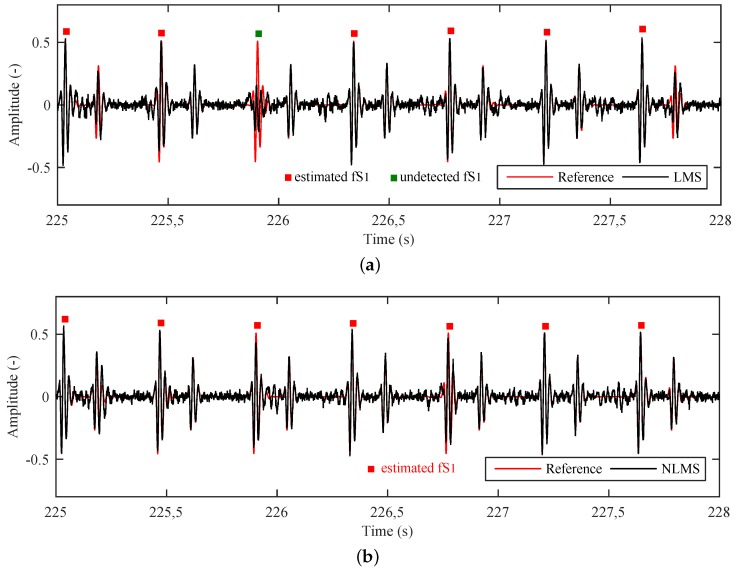
Detailed analysis of output from the adaptive system using (**a**) the LMS; and (**b**) the NLMS Algorithms.

Figure 15 shows a detailed comparison between the reference and filtered fPCG signals. It is evident that the filtered signal contains missing parts that negatively influences the detection of the necessary peaks for the determination of the fHR. This leads to a lower sensitivity and positive predicative value in fHR determination.

The value of $SNR_{inp}$ can be calculated by using the following equation:

(9) $$SNR_{inp}=10\log_{10}\frac{\sum_{n=1}^{N-1}{\left[sig_{usef}\left(n\right)\right]}^{2}}{\sum_{n=1}^{N-1}{\left[sig_{nois}\left(n\right)-sig_{usef}\left(n\right)\right]}^{2}},$$

where $sig_{usef}\left(n\right)$ is the desired signal (modelled reference time course of $IS_{T}$, i.e., fPCG) and $sig_{nois}\left(n\right)$ is the noise or unwanted signal (mPCG as measured in the abdominal region). In the software implementation described above, because the unwanted signal (noise) is formed by the addition of an ideal fPCG and $IS_{T}$ after their spread in the unknown environment of the human body, it is necessary to subtract the desired signal from the unwanted signal (noise) in the denominator of Equation (9).

The next filtering performance metric is the output $SNR_{out}$, which is defined by Equation (10). Based on $SNR_{inp}$ and $SNR_{out}$, we can determine how much the SNR value has improved ($SNR_{improv}$).

(10) $$SNR_{out}=10\log_{10}\frac{\sum_{n=1}^{N-1}{\left[sig_{des}\left(n\right)\right]}^{2}}{\sum_{n=1}^{N-1}{\left[sig_{predic}\left(n\right)-sig_{usef}\left(n\right)\right]}^{2}},$$

where $sig_{des}\left(n\right)$ is again the desired signal and $sig_{predic}\left(n\right)$ is a predicted (estimated) signal, or more precisely, the output from the adaptive filtering system. In Equation (9), it is necessary to subtract the desired signal from the predicted value in the denominator as the aim is to determine the noise signal that corresponds to the error signal.

By using the values of $SNR_{out}$ and $SNR_{inp}$, we can determine the efficiency of the implemented adaptive filters (Table 2 and Table 3). The prediction quality of these signal processing systems can be verified by the so-called predicative error, which shows the degree of inaccuracy between the predicted and the original output. In practice, we mostly use Mean Squared Error (further MSE) or more specifically the Root Mean Squared Error (RMSE) value defined by Equation (11).

(11) $$RMSE=\sqrt{\frac{1}{n}\sum_{i=1}^{n}{\left(sig_{des}-sig_{predic}\right)}^{2}}$$

To evaluate the peak detection success rate of our algorithms we can use two parameters. The first parameter is sensitivity, further labelled as S+, which shows what percentage of all peaks included in the examined signal were detected. The Sensitivity parameter S+ is defined by Equation (12).

(12) $$S^{+}\left[\%\right]=\frac{TP}{TP+FN}\cdot 100,$$

where TP is the number of correctly detected peaks (significant points) and FN is the number of undetected peaks.

The second parameter used for specifying the successful detection rate of peaks is the Positive Predictive Value—PPV. It is the probability of the real occurrence of a peak (significant point) during its positive detection by a detector. The PPV is expressed by Equation (13).

(13) $$PPV\left[\%\right]=\frac{TP}{TP+FN}\cdot 100$$

In Equation (13), TP is the number of correctly detected peaks and FP is the number of falsely detected peaks.

Table 2 and Table 3 tabulate the obtained results expressed in terms of quantitative measures when using the LMS and NLMS Algorithms, respectively. We carried out 10 independent experiments where we changed $SNR_{inp}$, which was dependent on the utilized model. In real measurements, $SNR_{inp}$ is influenced by the location of the sensor, the position of a fetus in the womb, gestation age, and other factors. Our experimental results demonstrate that the quality of signal filtering directly depends on $SNR_{inp}$.

The results (Table 2 and Table 3) show that the LMS Algorithm achieves better outcomes in terms of $SNR_{out}$ and RMSE metrics, while the NLMS Algorithm produces superior S+ and PPV values. This happens due to the fact that the LMS Algorithm very often misses some peaks or fS1s (Figure 15a) and consequently generates inferior S+ and PPV values even though it closely follows the reference signal. For more accurate fHR determination (Figure 15b), however, it is more suitable to use the NLMS Algorithm which, on the one hand, follows the reference signal less closely, but, on the other hand, does not miss signal peaks (fS1s) because it automatically adjusts the convergence constant μ (in contrast to what happens in the LMS Algorithm where no such adjustments are made).

## 4. Discussion

Fetal PCG monitoring is based on a passive acoustic technique (our fiber-optic interferometric sensors), where no energy is transmitted to the human body [20,119,120,121]. It offers clinically useful information that cannot be obtained with other methods such as ECG, MCG, Pulse Oximetry and Ultrasound. Various studies have proposed, extracted, and reported different parameters that carry useful diagnostic information about the fetus [122,123,124,125,126,127,128,129].

We had to use synthetic data in our research as noninvasive and nonintrusive sensors (like those used in our system) are not currently used in clinical practice: thus it was completely unrealistic to think about carrying out experimental tests using real maternal and fetal data to noninvasively detect fetal hypoxic conditions during different stages of pregnancy. For verification purposes, we used models that generate maternal and fetal signals with properties similar to real data acquired in clinical practice. These data also provided us with realistic reference signals for the quantitative evaluation of the realized systems, including the modelling of fetal hypoxic states without causing any risk or danger to the mother and her fetus.

To the best of our knowledge, this is pioneering work and the first study of its kind to explore the frontiers of noninvasive continuous fetal heart rate monitoring by means of fiber-optic interferometric sensors. We present a number of original and yet unpublished results. The follow-up research will be focused on the extension of our experiments and tests in clinical practice; at that time, measurements will be carried out on pregnant women. Fiber-optic sensors are resistant to technical artifacts such as electromagnetic interferences (EMI), and they can be used in Magnetic Resonance Imaging (MRI) environments or under wet conditions. The functionality of our sensor and adaptive filtering system was assessed by both qualitative (assessments made by gynecologists) and quantitative signal processing performance measures such as: SNR, RMSE, sensitivity S+, and positive predicative value PPV.

The basic drawback of fiber-optic interferometry is the need for a reference channel. The basis of interferometry is the measurement of differences between sensor and reference channels. The reference channel of the interferometer must be designed so that it allows to eliminate most of the unwanted signals. Thermal phenomena, mechanical changes, and other factors generate a background signal (noise). To address this issue or more precisely the need for the reference channel (position and encapsulation) is critical according to sensor sensitivity which must be taken into account in the design of a fiber-optic interferometer [56,130,131,132,133,134].

Even though the diagnostic potential of the fPCG signal is high, its processing and analysis are rather challenging. Fetal heart sound (fHS) signals could be relatively weak and have a low SNR due to the following factors: the uncertainty associated with the presence of the $S_{2}$ component of the fetal heart sounds in the de-noised fPCG signal [109], the proximity of fHS signals to the threshold of audibility, which may lead to problems of degree and accuracy [135], the spread and attenuation of generated heart sounds through a time-varying transmission path (made up of amniotic fluid, the uterine muscular wall, bones, cartilage and layers of fat tissue). At the boundaries of each of these layers, heart sounds are attenuated, reflected, and refracted mainly due to the impedance mismatch between layers and signal scattering [136]. As a consequence signal dampening will occur and fetal heart sounds will have a very narrow band of frequencies [127,128].

The next stage of research activity within the framework of this paper is to carry out animal studies (preferably using a pig model as sheep and calf models are not generally suitable [137] for this purpose). Clinical trials will be planned by leveraging our collaborative arrangements made with the Department of Obstetrics and Gyneacology, at the Masaryk University and University Hospital in Brno in the Czech Republic.

We express our readiness to carry out a detailed analysis of the applicability of patch monitors to fHR monitoring. We are aware that wearable ’electronic patch monitors’ [138,139] are great candidates for wireless continuous monitoring of biopotentials representing vital signs. In our most recent research (submitted for publication) we specifically focused on signal processing techniques applicable to the fetal electrocardiograms (fECGs) filtered from the abdominal (aECG) signals. Suppressing the unwanted components (especially the maternal ECG) from the aECG signal proved to be a very challenging task. In comparison to fECGs, fetal Phonocardiography, which is based on sensing the fetal heart sounds on the maternal abdomen, produces a fetal PCG (fPCG) component, which is significantly larger in amplitude than the fECG component in the abdominal ECG (aECG). Therefore, it is easier to extract. For this purpose, we decided to utilize optical fibers, which are frequently used in many other applications. The many other advantages of our proposed approach are described in our paper.

## 5. Conclusions

This paper deals with the design and implementation of a noninvasive fiber-optic sensor and its associated adaptive signal processing system for fetal heart rate (fHR) monitoring. The probe utilizes sensing elements operating on the basis of the Mach-Zehnder Interferometer, which offers great potential for applications in modern noninvasive medicine. The performance of our system was evaluated by using generated (synthetic) data comparable to real data with the added advantage of providing the reference signal necessary for the performance evaluation of the adaptive filtering system as well as the capability to model hypoxic states without causing any risk or danger to the fetus and the mother. The system’s performance was evaluated by using both qualitative (gynecological expert knowledge and experience) and quantitative measures such as SNR, RMSE, S+, and PPV.

Our experimental results demonstrated that the NLMS Algorithm produced better outcomes in the determination of fHR information based on S+, PPV measures, whereas the LMS Algorithm performed better when SNR and RMSE parameters where used to evaluate the system’s performance. Our findings further confirmed that signal filtering efficiency was not influenced by the form of fPCG signals (physiological or pathological) when evaluated based on SNR, RMSE, S+, and PPV parameters.

In summary, our paper presents the first attempt of its kind towards developing the techniques and methods for noninvasive continuous fHR monitoring by using interferometric fiber-optic sensors. The area of research dealing with fetal PCGs measured by these sensors is an uncharted territory that warrants further extensive clinical investigations to be carried out in the future.

Our realized noninvasive adaptive system using fiber-optic interferometric sensors can significantly contribute to the future research in fHR monitoring and can even be considered for application in the Magnetic Resonance Imaging (MRI) and wet environments. We are very excited that the clinicians at the University Hospital, Brno, in the Czech Republic, intend to use the system reported here for their short term clinical studies in the near future. 

## Figures and Tables

**Table 1:** Summary of main methods used for the noninvasive electronic fetal heart rate monitoring.

Method	Technical Specification	Gestational Age (GA)	Pros and Cons
CTG	2 transducers—measurement of fHR and uterine activity	≥20 weeks	− Ultrasound radiation − No information about BTB variability + Smooth HR in a time line + Rather robust and reliable + Measures uterine contractions + Cheap
fMCG	Detection of fetal magnetic field SQUID Sensors placed near maternal abdomen	≥20 weeks	− Expensive − Needs trained staff − Easy morphological analysis due to higher SNR
NI-fECG	Standard ECG electrodes	≥20 weeks with DIP from 28th to 37th week	+ Quite accurate + Safe + Easy to use + Continuous monitoring of fHR + future possibility of morphology of low SNR − Susceptible to technical artifacts (e.g., network noises)

**Table 2:** Experimental result when using the LMS Algorithm.

${\mathrm{SNR}}_{\mathrm{inp}}\;(\mathrm{dB})$	${\mathrm{SNR}}_{\mathrm{out}}\;(\mathrm{dB})$	RMSE(mV)	$S^{+}\;\left(\%\right)$	PPV(%)
−9.1497	1.8467	0.0289	95.7145	98.5401
−3.6984	2.1514	0.0317	96.4967	98.5417
−5.9841	3.4791	0.0497	97.1574	98.6053
−4.1385	0.2001	0.0304	91.5781	97.9471
−6.4717	−2.7197	0.0241	87.7339	96.5812
−4.2581	2.0964	0.0301	96.1987	98.3271
−7.5717	2.9417	0.0431	96.7417	98.5981
−8.1741	2.0114	0.0291	96.2852	98.4179
−3.1741	3.1314	0.0478	96.9517	98.3271
−10.3947	−0.3971	0.0209	89.4719	96.9547

**Table 3:** Experimental results when using the NLMS Algorithm.

${\mathrm{SNR}}_{\mathrm{inp}}\;(\mathrm{dB})$	${\mathrm{SNR}}_{\mathrm{out}}\;(\mathrm{dB})$	RMSE(mV)	$S^{+}\;\left(\%\right)$	PPV(%)
−9.1497	1.5414	0.0234	97.8714	98.8475
−3.6984	1.9771	0.0287	97.0167	98.1394
−5.9841	3.2419	0.0432	98.8471	98.8417
−4.1385	0.2141	0.0217	95.6717	98.6614
−6.4717	0.0187	0.0281	95.1423	97.7491
−4.2581	1.8874	0.0279	97.5140	98.3405
−7.5717	2.5173	0.0407	98.3524	98.6477
−8.1741	1.7491	0.0259	98.1415	98.3069
−3.1741	2.6524	0.0427	97.9146	98.7421
−10.3947	0.0350	0.0185	95.0297	97.8140
